# Paying for the quantity and quality of hospital care: the foundations and evolution of payment policy in England

**DOI:** 10.1186/s13561-015-0050-x

**Published:** 2015-06-12

**Authors:** Katja Grašič, Anne R. Mason, Andrew Street

**Affiliations:** Centre for Health Economics, University of York, York, YO10 5DD UK

**Keywords:** Diagnosis-related groups, Healthcare resource groups, Prospective payment system, Reimbursement mechanisms, Benchmarking, England

## Abstract

Prospective payment arrangements are now the main form of hospital funding in most developed countries. An essential component of such arrangements is the classification system used to differentiate patients according to their expected resource requirements. In this article we describe the evolution and structure of Healthcare Resource Groups (HRGs) in England and the way in which costs are calculated for patients allocated to each HRG. We then describe how payments are made, how policy has evolved to incentivise improvements in quality, and how prospective payment is being applied outside hospital settings.

## Introduction

The diversity and complexity of hospital care makes it challenging to devise reimbursement arrangements that ensure that the amount and quality of hospital care meets the needs of the population yet remains affordable. Most countries have adopted some form of prospective payment to encourage efficient provision of care, differentiating payments using local variants of Diagnosis Related Groups (DRGs) such as the Healthcare Resource Groups (HRGs) used in England. In this article we describe the evolution and structure of HRGs in England and the way in which costs are calculated for patients allocated to each HRG. We then explain how payments are made, how policy has evolved to incentivise improvements in quality and how prospective payment is being applied outside hospital settings.

## Review

### Development of the HRGs

The origins of HRGs can be traced back to 1981, when the Department of Health commissioned research to assess the ability of North American DRGs to explain variation in the length of stay of English patients [1]. After a first refined version of the US DRG system was created in 1987, the United Kingdom’s own categorization system of HRGs was launched in 1991 [1]. While DRGs were based on major diagnostic categories (MDCs) that correspond to a single organ system, HRGs are more directly related to specialties (Table 1) and draw upon national procedure codes, developed by the Office of Population Censuses and Surveys (OPCS),^a^ in addition to the International Classification of Diseases (ICD) codes for diagnoses.

**Table 1:** HRG root structure

Chapter	Chapter Description
A	Nervous System
B	Eyes and Periorbita
C	Mouth Head Neck and Ears
D	Respiratory System
E	Cardiac Surgery and Primary Cardiac Conditions
F	Digestive System
G	Hepatobiliary and Pancreatic System
H	Musculoskeletal System
J	Skin, Breast and Burns
K	Endocrine and Metabolic System
L	Urinary Tract and Male Reproductive System
M	Female Reproductive System
N	Obstetrics
P	Diseases of Childhood and Neonates
Q	Vascular System
R	Radiology and Nuclear Medicine
S	Haematology, Chemotherapy, Radiotherapy and Specialist Palliative Care
U	Undefined Groups
V	Multiple Trauma, Emergency Medicine and Rehabilitation
W	Immunology, Infectious Diseases and other contacts with Health Services
X	Critical Care and High Cost Drugs

*Source*: National Casemix Office, 2014 [35]

The first version of HRGs comprised 534 categories (including 12 ‘undefined’ categories: these reflect coding quality issues, for example missing primary diagnosis or age) but did not cover all acute activity, lacking groups for psychiatry, radiotherapy and oncology [2]. HRG version 2 was released in 1994, comprising 533 categories, including six undefined (‘U’) groups, but now including psychiatric HRGs. Further refinements led to the release of HRG3.1 in 1997, comprising 572 groups and including chemotherapy [3]. Another revision appeared with the release of HRG3.5 in 2003, expanding the number of groups to 610.

The HRG4 design represented a major development from HRG3.5 in two key respects. First, under HRG3.5, each episode of care generated a single core HRG. Under HRG4, some high-cost elements of treatment were separated from the core-HRG, generating ‘unbundled’ HRGs. Unbundled HRGs capture eight broad types of specialised care^b^ that may be provided in different ways, in different settings or by different providers [4]. Second, the number of HRGs more than doubled, with coverage expanding to include non-admitted (outpatient) care, emergency medicine and some specialty areas not covered by HRG3.5, such as critical care [5].

HRG4 was first used in the 2006/07 reference cost collection exercise and replaced HRG3.5 as the basis for reimbursement in 2009/10 [6].

HRG4 was designed to evolve year on year, but in 2012/13 a more extensive update, referred to as HRG4+, provided even greater differentiation for complications and co-morbidities [7]. The additional HRG codes were mostly created by granulating existing HRGs into several splits that better reflect complications and comorbidities and are therefore more suitable for distinguishing cases with high-resource use, reflected either by higher cost or longer length of stay. HRG4+ is being introduced in three phases from 2012/13, each phase involving refinements to a subset of HRGs.

### Use of HRGs

The application of the HRG system has evolved over time [8]. When first introduced, HRGs were used for benchmarking, providing the basis for comparative performance assessment and commissioning. Hospitals could use an interactive national database to compare length of stay for their patients in an HRG against the national average or against a selection of hospitals. Subsequently, hospitals started to use HRGs for internal resource management, to monitor actual versus expected expenditure, and to assess the budgetary impact of anticipated changes in the volume and casemix of patients within specialties or clinical directorates.

By the late 1990s, HRGs were being used for contractual purposes. At that time hospitals received their income via three main types of contractual arrangement. Block contracts specified payment for a fixed volume of activity; cost-and-volume contracts allowed for payments to be withheld (or made) if volume levels were below (or surpassed) expectations; and cost-per-case contracts involved patient-specific payments. Originally, contracts distinguished patients according to the specialty in which they were treated but, from 1994 onward, increasingly more contracts were specified using HRGs.

Announced by the Labour government in 1997, a national schedule of ‘reference costs’ was developed itemizing the cost of HRGs across the NHS [9]. Benchmarking costs in a standardized manner enabled purchasers to identify cost inefficiency. However, without information about case-mix and outcomes, the provision of cost information alone was probably an insufficient incentive for hospitals to take action to address cost differentials [10].

In 2002, the Government published proposals to introduce a prospective payment system, with hospitals receiving a fixed national payment per patient depending on the HRG to which they were allocated [11]. Payment by Results (PbR)—as these reimbursement arrangements were called—was introduced for a small number of HRGs in 2003/4, and coverage gradually expanded to other HRGs.

In 2013/14, PbR was superseded by the National Tariff Payment System [12] which extended these prospective payments beyond hospital care to NHS healthcare services more generally [12]. Table 2 shows the evolution of the HRG system, including changes in the numbers of categories and scope.

**Table 2:** Overview of the evolution of the English HRG system

	1st DRG version	2nd DRG version	3rd DRG version	4th DRG version	5th DRG version	6th DRG version
Date of introduction	May 1992	August 1994	June 1997	October 2003	October 2006	Phase 1: April 2013
(Main) Purpose	Patient classification	Patient classification	Patient classification	Patient classification, reimbursement	Patient classification, reimbursement	Patient classification, reimbursement
DRG system	HRG1	HRG2	HRG3.1	HRG3.5	HRG4	HRG4+
Cost and/or performance data used for development	Adaptation of United States DRGs	Data analysis of groupings	Clinical review to refine for ICD-10. Statistical analysis	Clinical Working Groups refined categories.	Expert working groups’	Expert working groups, clinical communities, as well as international casemix developments and best practice*
					micro-costing data	
				Statistical analysis		
Number of DRGs	534	533	572	610	Updated annually:	Updated annually:
					1389 (2006/7) to 1657 (2011/12)	2100
Applied to	Public hospitals	Public hospitals	Public hospitals	Public hospitals/private hospitals or treatment centres treating NHS patients	Public hospitals/private hospitals or treatment centres treating NHS patients	Public/private hospitals or treatment centres treating NHS patients
	Acute admissions	Acute admissions	Acute admissions	Acute admissions	Acute admissions	Acute admissions
					Outpatients	Outpatients
					Critical Care	Accident and Emergency
						Critical Care

*Developed in partnership with the clinical community, as represented and endorsed by the Royal Colleges Associations and Professional Bodies. The increased applicability of the Casemix classification to emerging policy requirements has been influenced by findings from the Department of Health's International Review of Classifications, as well as international casemix developments and best practice

*Sources*: Anthony, 1993 [2]; Benton, 1998 [3]; Casemix Design Authority, 2009 [4]; NHS Information Centre for Health and Social Care, 2008 [5]; Information Standards Board for Health and Social Care, 2009 [6]; National Casemix Office, 2013 [7]; National Casemix Office, 2014 [35]

### Structure of HRG4+

**Fig. 1:**
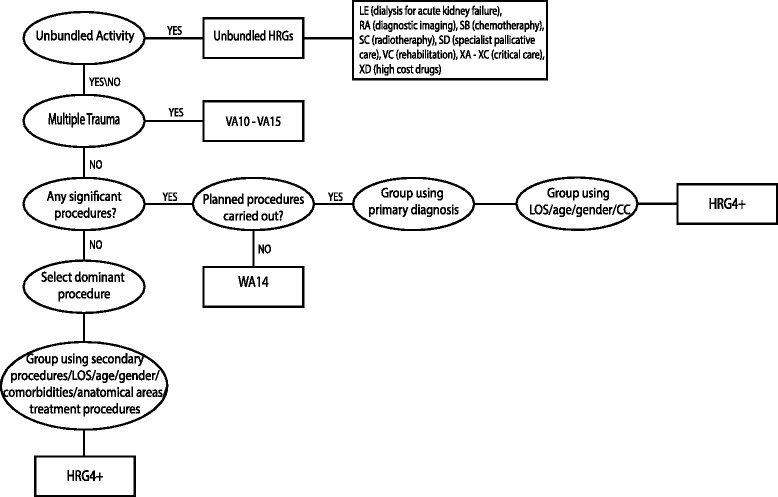
HRG4–Classification flow chart for inpatients. *Sources*: Code to Group Worksheet, HSCIC

HRGs are designed to be clinically similar and resource homogeneous [2]. Several different approaches are in use for the design and sense checks of the classification system, among them (but not limited to) Classification and Regression Trees (CART), Reduction in Variance (RIV) and Minimum Volume Ellipsoid [13]. These methods allow for identification of outliers and differentiation between patients with high or low resource use. Patients are allocated to HRGs on the basis of information in their electronic medical record using grouping software [12], which is available online.^c^ If any of the required data fields are missing or invalid, the patient is allocated to an ‘error code’ HRG. The stages of the grouping algorithm for HRG4+ are shown in Fig. 1.

*Unbundling* is the first step in the grouping process [14], whereby some particular high cost procedures, diagnostic imaging and high cost drugs are allocated to separate ‘unbundled’ HRGs. The grouper then ignores these unbundled components when deriving the core HRG for each patient. Unbundling elements of ‘event-based’ care from the core-HRG provides greater scope for services to be provided in non-inpatient settings where appropriate.

The second step involves identification of high-resource, complex treatments associated with *multiple trauma* sites. A patient is assigned a multiple trauma HRG if the treatment involves at least two specific body sites.

The third step involves ranking *procedures* using a hierarchy based on cost data and clinical knowledge. Where several procedures are recorded, the procedure with the highest hierarchy value determines the HRG allocations [15]. In case of multiple procedures with the same hierarchy value, the one listed first in the medical record is used for grouping. If procedures are planned but not carried out, patients are allocated to a specific HRG (WA14).

If no procedure with a hierarchy value of 5 or more is recorded, the HRG is assigned using diagnosis hierarchies. This follows the same steps as grouping using procedure values.

Complication and comorbidity (CC) splits are a way of incorporating variations in severity and complexity within HRGs. Lists of CC splits are specific to each HRG chapter and are usually based on diagnosis codes. Some HRGs are also split by procedures, age, length of stay, anatomical region or treatment approach. In HRG4+, CC splits are based on the summed ‘score’ of all comorbidities present [15].

**Fig. 2:**
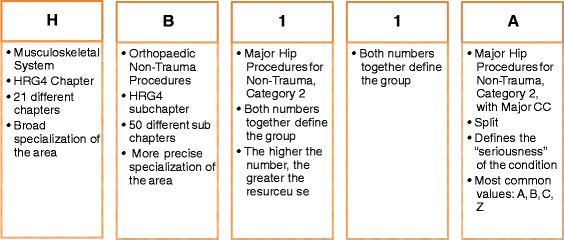
Composition of HRG code HB11A (Major Hip Procedures for Non-Trauma, Category 2, with Major CC). *Sources*: Code to Group Worksheet, HSCIC

Each HRG4+ code is composed of five alphanumeric characters (AANNA). The first letter represents one of 21 chapters and the second letter defines the sub-chapter, narrowing down the treatment area (see Fig. 2). The next two characters represent the number within the chapter/sub-chapter; in general, lower numbers indicate higher expected resource use [15]. The final letter defines the split or level of severity within the HRG. Usual splits are ‘A’, ‘B’ and ‘C’, where ‘A’ is usually (but not always) an indicator of greater resource use. The letter ‘Z’ indicates that the HRG has no splits.

### Costing of HRGs

All NHS hospitals are required to report their activity and unit costs annually to the Department of Health [16]. The rules for costing are updated on a regular basis and are summarised in *Approved Costing Guidance* [17]. Currently, the mandatory reporting of costs is using a top-down approach, although efforts are in place to motivate providers to report their costs at patient level, using Patient-Level Information and Costing Systems (PLICS).

Top-down costing requires that unit costs reflect the full cost of provision and include all operating expenses, staff costs and capital costs (both interest and principal), but exclude the costs of teaching and research. The starting point for the top-down costing process is the general ledger. Here, total costs or ‘high-level control totals’ are established. Aggregate costing figures are then divided into one of three cost categories: direct, indirect and overheads. Direct costs are those which can be directly attributed to the service(s) that generated them. For instance, the type and amount of nursing staff working in a particular specialty can be estimated with reasonable precision.

Costs that cannot be attributed directly must be apportioned by other means. Indirect and overhead costs are pooled in order to do this.^d^ These ‘cost pools’ bring together costs into identifiable groups (for example, wards, pharmacies, theatres) which are then apportioned to the relevant departments. These allocations take account of the fixed, semi-fixed or variable^e^ nature of the resource in question.

**Fig. 3:**
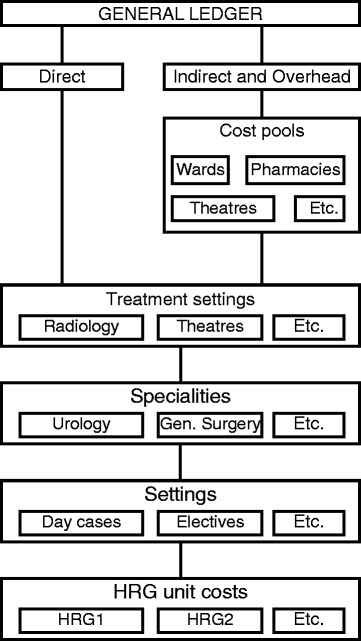
English cost-accounting system. *Sources*: Department of Health, 2009 [24]; Healthcare Financial Management Association, 2012 [34]

Fig. 3 illustrates stages of this costing exercise [16].

The next step involves allocations to treatment settings (e.g. theatres, radiology) and specialities (e.g. urology, general surgery). This allocation may be direct (e.g. wages of nurses working on a particular ward) or indirect (e.g. cleaning costs of theatres or wards). Costs are then allocated according to the point of delivery, indicating whether the patient was treated as a day case or as an elective, non-elective or maternity inpatient, in an outpatient (ambulatory) department, or in ‘other’ settings.^f^

Finally, costs are allocated to HRGs, taking account of the volume of patients in each HRG in each setting and key cost drivers including length of stay or the number of prostheses used. The outcome of this cost-allocation process is a cost per HRG according to the type of admission for each hospital specialty.

For each HRG there will be a small number of cases which have an abnormally long length of stay. An upper trim-point is calculated for each HRG: the upper quartile of the length of stay distribution for that HRG plus 1.5 times the interquartile range [18]. A cost per excess bed day is calculated for patients that stay beyond the trim point.

### Calculation of HRG prices and form of payments

Currently, most acute hospital care in England is reimbursed under the prospective payment system now termed ‘the National Tariff Payment System’ and administered by Monitor, the independent regulator for health services [12]. In 2014/15, national tariffs were payable for most admitted patient care, outpatient care and A&E services. However, there remained scope for variation from national tariffs, allowing commissioners and providers to agree local prices for some types of activity, such as for high-cost drugs, magnetic resonance imaging (MRI) scans, cochlear implants, orthopaedic prostheses and chemotherapy [12].

The national tariff is determined for the year ahead by the Department of Health according to a standard methodology [19]. Details of the tariffs for admitted patients, outpatients and A&E attendances are summarized in Table 3. Prices are set based on the average of the costs calculated by all hospitals for each of their HRGs.

**Table 3:** Payment arrangements, 2014/15

	Admitted patients	Outpatients	A&E	Post discharge rehabilitation	Unbundled HRGs
Currency	HRG spell	Treatment function code (TFC): attendance by specialty	HRG Attendance	Bed days	Events
		HRGs: for procedures			
Structure	Tariffs for:	Tariffs for:	Tariffs vary by:	Tariffs for 4 types of post discharge rehabilitation:	Chemotherapy
	• electives & day cases	• first attendance	• Type of investigation	• Cardiac	• a core HRG (covering the primary diagnosis or procedure) –national price
	• non-electives	• follow-up attendance	• Category of treatment	• Pulmonary	• unbundled HRGs for chemotherapy drug procurement—local currencies and prices
	• short-stay elective	• multi-professional/single professional appointments	• Provider type	• Hip replacement	• unbundled HRGs for chemotherapy delivery—national prices
	• short-stay emergencies (>2 days)	• separate national prices for diagnostic imaging		• Knee replacement	Radiotherapy:
	• Best practice tariffs	Procedures carried out in outpatient setting subject to non-mandatory tariff based on HRGs		National prices to shift responsibility for patient care following discharge to the acute provider who treated the patient. Applicable only where a single trust provides both acute and community services.	• unbundled HRGs for planning and treatment—national or local prices
	• Pathway payments	Non-mandatory tariff for outpatient appointments not carried out face to face			
	o Maternity care				
	o Cystic fibrosis				
	Long-stay outlier payment triggered at predetermined length of stay (dependent on HRG).				
Specialized service adjustments	Best practice tariffs for 17 types of care	Local prices for outpatient attendances that are not pre-booked or consultant-led.	Type 3 A&E departments are eligible for the simplest currency only		
	Top-up payment for specialized services for children, spinal surgery, neurosciences and orthopaedic activity		NHS Walk-in Centres are paid by local prices, not by the tariff		
Rules and Flexibilities	Unbundling: see column 5	Unbundling of care pathway subject to local agreement	Local flexibilities could be applied to support service redesign		
	Emergency admissions: the marginal rate emergency rule				
	Emergency readmissions: the 30 day emergency readmission rule				

*Sources*: Monitor 2013 [12]; Department of Health, 2009 [24]

Note: Teaching and research are funded entirely separately, and their costs are not included in the national tariff. ‘Currency’ is the unit of payment

The base tariff for each HRG (*i* = 1… I) and admission type (*j* = 1…5) for a given year *t*, *p*_*ijt*_, is calculated as:

1 $$ {p}_{ijt}={\pi}_i{\overline{c}}_{ijt-3} $$

where ${\overline{c}}_{ij}$ is the average cost for each HRG by admission type across all hospitals. There is a three-year^g^ delay between hospitals submitting cost data and these data being converted into prices, hence the *t*-3 subscript attached to these average costs. To take account of this delay, an adjustment π_*i*_ is made to each HRG. This adjustment is HRG-specific, allowing for inflationary impacts such as clinical guidance and technology appraisals issued by the National Institute for Health and Care Excellence (NICE) that may have occurred in the intervening period and for improvements in efficiency [21]. An efficiency factor of 3.8 % was set for 2015/16, and many hospitals initially rejected the proposed tariff arrangements [22]. After a period of negotiation between Monitor and hospitals, 88 % of hospitals accepted the so-called enhanced tariff option (ETO) for 2015/16. Those that did not continued to be paid on the basis of 2014/15 tariffs.

HRG-specific per diem payments are made if patients stay in hospital beyond HRG specific length of stay trimpoints. The excess bed day costs reported by hospitals are used to calculate these payments.

While a single national tariff applies, it is recognised that some costs relating to labour, land and buildings are outside the control of hospitals. The overall impact of these exogenous costs is corrected by the Market Forces Factor (MFF).^h^ In the past, the MFF was paid directly by the Department of Health, but purchasers (clinical commissioning groups, known as CCGs) now make the MFF payments at the same time as activity payments [24].

Top-up payments are also made for specialised services, in recognition that cost differences may not be adequately captured by HRGs [25]. In 2014/15, specialist top-ups were made for provision of specialised care for children (top-up: 44 to 64 per cent), neurosciences (28 per cent), and spinal surgery (32 per cent) and orthopaedics (24 per cent) [12].

Finally, to incentivise lower rates of emergency admissions and to encourage providers and commissioners to work together to reduce the demand for emergency care, acute hospitals are paid 30 per cent^i^ of the national tariff for increases in the value of emergency admissions above an agreed baseline [12]. Commissioners must spend the remaining 70 per cent on managing demand for emergency services.

The tariff system has driven the development of classification systems for care delivered in non-hospital settings. The scope of the payment system has been progressively extended to cover adult mental health, long-term conditions, preventative services, sexual health, community services, ambulance services and out-of-hours primary care [26]. The work on adult mental health is to be extended to cover psychological therapies (Improving Access to Psychological Therapies-IAPT), children’s and adolescent mental health, forensic mental health, learning disabilities and liaison psychiatry [12].

New currencies for palliative and end of life care aim to describe differences in the complexity and cost of patients in need of palliative care. The currencies have been defined using data collected through 11 Palliative Care Funding Pilots that ran between July 2011 and April 2014 and have been in (non-mandatory) use since 2015/16. Twenty-eight adult and 28 children currencies are intended for use in acute, community care and hospice setting and are built around four phases of illness: stable, unstable, deteriorating and dying [27].

### Quality-related adjustments

From 2009/10, all acute trusts have been required to publish ‘quality accounts’ alongside their financial accounts [28]. The Commissioning for Quality and Innovation (CQUIN) payment framework came into effect in April 2009. It allows commissioners to link a specific, modest proportion of providers’ income to the achievement of realistic locally agreed goals. Examples of local goals set in 2012/13 include provision of smoking cessation support, improvement of hospital discharge/clinical communication, promotion of better responsiveness to personal needs of patients and improvement of hospital food. The CQUIN payment framework originally covered 0.5 per cent of a provider’s annual contract income [29] and this rose to 2.5 per cent in 2014/15 [30]. There are also four national CQUINs, selected on a yearly basis that aim to incentivise both quality and efficiency by creating new patterns of care; in 2014/15 they comprised patient experience (Friends and Family Test), dementia and delirium care, reduction of harm (NHS Safety Thermometer), and improving physical healthcare for people with severe mental illness [30].

An important development is the introduction of ‘best practice’ tariffs’ (BPTs) for high-volume areas that are characterised by significant levels of unexplained variation in quality of clinical practice and for which there is clear evidence of what constitutes best practice [31]. The tariffs reflect the costs of delivering best practice and are intended to incentivise a shift away from ‘usual care’, which is reimbursed by the standard HRG tariff. The selection and development of BPTs depends on evidence of variation in practice as well as on feasibility of collecting high quality data. For example, the Institute for Innovation and Improvement found that, in 2005/6, the national average day case rate for cholecystectomies was just 6.4 % and there were significant variations across hospitals in the proportion of the procedures undertaken laparoscopically, in length of stay and in the day case rate. The optimal ‘pathway of care’ for cholecystectomy and recommendations for its delivery were then designed based on a literature review, site visits, and semi-structured interviews [32].

The impact of individual BPTs is variable and in some cases BPTs were not themselves considered to be the driving force for local improvement [32]. Nevertheless, some areas have shown significant improvement; for example, only 37 % of eligible patients were given the BPT uplift for hip fracture care at the beginning of 2011 and this rose to 64 % in the last quarter of 2013 [33].

Table 4 provides an overview of the development of BPTs, including a ‘year of care’ capitation payment for outpatient services in paediatric diabetes, and pathway payments for maternity and cystic fibrosis services. There are plans to develop capitation payments for those with long-term conditions, and new currencies for palliative and end-of-life care [12].

**Table 4:** Introduction and development of best practice tariffs

**2010/11**	Cataracts	Aims to reduce the number of times patients are assessed before and after surgery by setting a price for the whole pathway rather than pricing each spell of activity; the pathway should be in line with recommendations provided by Royal College of Ophthalmologists
	Cholecystectomy (gall bladder removal)	Encourages keyhole surgery in a day case setting where clinically appropriate
	Fragility hip fracture	Makes an additional payment for providing rapid surgery and orthogeriatric care
	Stroke	Makes additional payments for urgent brain imaging and care in an acute stroke unit.
**2011/12**	Adult renal dialysis	Aims to improve care for patients undergoing haemodialysis
	Day case procedures	Encourages providers to increase their day case rates in a number of surgical procedures including hernia repair and prostate resection; by 2014/15 fifteen high volume procedures are included in the tariff.
	Interventional radiology	Incentivises use of minimally invasive techniques rather than open surgery where clinically appropriate; in 2014/15 seven procedures are included in the Best Practice Tariff programme
	Paediatric diabetes	Aims to improve quality of diabetes care; from 2014 includes also inpatient stays for young people with diabetes
	Primary total hip and knee replacements	Encourages best clinical management of patients and reductions in length of stay
	Transient ischaemic attack (or mini-stroke)	Paid for timely and effective outpatient systems for treating patients with TIA
**2012/13**	Major trauma	Encourages best practice treatment and management of trauma patients within a regional trauma network; in 2014/15 there was a change in best practice criteria
	Same day emergency care	Promotes management of 12 clinical scenarios on a same day basis in an ambulatory emergency care manner
	Procedures in outpatients	Encourages three procedures (diagnostic cystoscopy, diagnostic hysteroscopy and hysteroscopic sterilisation ) to be performed in an outpatient setting
	Paediatric diabetes	Applies to providers who provide services in accordance with the best practice specification
**2013/14**	Early inflammatory arthritis	Services must meet four criteria, dealing with early referral and treatment start as well as regular subsequent appointments
	Endoscopy procedures	Encourages providers to meet quality standards in line with the |Joint Advisory Group accreditation scheme for endoscopy services.
	Paediatric epilepsy	Intended for follow up appointments
	Parkinson’s disease	Aims to reduce waiting time for treatment
	Pleural effusions	Applies to unilateral effusions and increasing use of thoracic ultrasound.
**2014/15**	Hip and knee replacement	Payments linked to patient reported outcome measures (PROMs)

Sources: Department of Health, 2013 [18]; Monitor, 2013 [12]

## Conclusions

Creating an efficient, fair and transparent funding model for healthcare is a dynamic process, as it is influenced by technological advancements, new policies and change in population demographics. There have been several major overhauls of the HRG system over the last three decades, as well as annual revisions. In this article we have described the evolution and structure of HRGs in England, the way in which costs are calculated for patients allocated to each HRG, and how HRGs underpin the prospective payment system. HRGs have evolved from a means of classifying activity, then to paying for activity, and to incentivizing quality and better outcomes for patients, both within and beyond hospital settings.

It is likely that HRGs will be further granulated to adjust for the more difficult cases and in response to technological changes. This is already evident in the development of the HRG4+ system, with new currencies added on a yearly basis, covering a wide range of activities in different settings. It is also likely that best practice tariffs will be extended to other areas, so that payments become more outcome-focused and not just activity-based. There may also be greater interest in currencies based on care pathways, already introduced for mental health and palliative care, as these potentially incentivise integrated care based on patient need rather than incentivising activity. These welcome directions of travel represent the next challenge for policy development and evaluation over the coming decade.

### Endnotes

^a^OPCS 4.7 was implemented in April 2014

^b^Chemotherapy; critical care; diagnostic imaging; high cost drugs; radiotherapy; rehabilitation; specialist palliative care; renal dialysis for acute kidney injury.

^c^The Payment Grouper for 2014/15 is available from: http://www.hscic.gov.uk/article/3938/HRG4-201415-Payment-Grouper [previous years are available in the archive]

^d^dIndirect costs are indirectly related to the delivery of patient care, but cannot always be specifically identified to individual patients. Overhead costs are the costs of support services that contribute to the effective running of an NHS provider. These costs cannot be traced or easily attributed to patients, and need to be allocated via an appropriate cost driver [17].

^e^Fixed costs are those that do not change as activity changes (e.g. annual contract cost for cleaning services). Semi-fixed costs are those that do not change with small changes in activity but that ‘step up’ when a certain threshold is reached (e.g. nursing staff). Variables costs are those that are directly affected by the number of patients treated or seen (e.g. drug costs) [17].

^f^‘Other’ here refers to all other hospital costs that are not part of day-case, inpatient or outpatient activity. It includes community services, critical care services, A&E medicine, radiotherapy and chemotherapy, renal dialysis, and kidney and bone marrow transplantation, for example.

^g^Prior to the Lawlor review there was a two-year lag [20].

^h^For a description of the methods for calculation of Market Force Factors, see reference [23].

^i^From 2015/16: 70 % under the new enhanced tariff option (ETO) https://www.england.nhs.uk/2015/03/06/eto-2015-16/. This money, which would otherwise have been spent by CCGs on admission avoidance measures, is now available to providers to be invested in acute services, including but not limited to winter resilience schemes [2015/16 tariff arrangements FAQ)

## Abbreviations

A&E: Accident & Emergency
BPT: Best practice tariff
CART: Classification and regression tree
CC: Complication and comorbidity
CCG: Clinical Commissioning Group
CQUIN: Commissioning for Quality and Innovation
DRG: Diagnosis Related Group
HRG: Healthcare Resource Group
IAPT: Improving access to psychological therapies
ICD: International Classification of Diseases
MDC: Major diagnostic category
MFF: Market forces factor
MRI: Magnetic resonance imaging
NHS: National Health Service
NICE: National Institute for Health and Care Excellence
OPCS: Office of Population Censuses and Surveys
PbR: Payment by results
PLICS: Patient-level information and costing systems
RIV: Reduction in variance
TFC: Treatment function code
